# Post SARS-CoV-2 infection reactive arthritis: a brief report of two pediatric cases

**DOI:** 10.1186/s12969-021-00555-9

**Published:** 2021-06-12

**Authors:** Reza Sinaei, Sara Pezeshki, Saeedeh Parvaresh, Roya Sinaei, Reza Shiari, Mehrnoush Hassas Yeganeh, Nasrin Bazargn, Nava Gharaei

**Affiliations:** 1 Department of Pediatrics, School of Medicine, Kerman University of Medical Sciences, Kerman, Iran; 2 Endocrinology and Metabolism Research Center, Institute of Basic and Clinical Physiology Sciences, Kerman University of Medical Sciences, Kerman, Iran; 3 Department of Internal Medicine, School of Medicine, Kerman University of Medical Sciences, Kerman, Iran; 4 Department of Pediatric Rheumatology, School of Medicine, Shahid Beheshti University of Medical Sciences, Tehran, Iran; 5 Shahid Beheshti University of Medical Sciences, Tehran, Iran; 6 Department of Pediatrics, Afzalipour Medical Center, School of medicine, Kerman University of Medical Sciences, Kerman, Iran; 7 Department of Molecular and Cellular Biology, Harvard University, Cambridge, Massachusetts USA

**Keywords:** COVID-19, SARS-CoV-2, Limping

## Abstract

**Background:**

Although, preliminary reports of Severe Acute Respiratory Syndrome (SARS)-CoV-2 infection suggest that the infection causes a less severe illness in children, there is now growing evidence of other rare or even serious complications of disease.

**Case presentation:**

During the recent COVID-19 pandemic in Kerman, Iran, two children (an 8 year-old boy and a 6 year-old girl) were referred to outpatient Clinic of Pediatric Rheumatology with complaints of limping. Both children had experienced fever and mild respiratory tract infection. At the beginning of the second week of infection, they developed joint effusion. They both tested positive for coronavirus infection and were therefore diagnosed with post Coronavirus reactive arthritis. Both children were treated successfully with rest and Non-Steroidal Anti-Inflammatory Drugs (NSAID). They did not have any medical problems in the two months fallow up.

**Conclusions:**

These two cases suggest that COVID-19 may be rheumatogenic. Highlighting the need for awareness of physicians, especially pediatricians, regarding the pathogenesis margins of this virus, as late presentations are of great importance.

## Background

Limping is a deviation from a normal age-appropriate gait pattern. It is caused by various conditions and is a common cause of referrals to pediatric Rheumatology and Emergency Clinics [1]. The incidence rate of limping in children has been suggested to be around 1.8 per 1000 children [2]. While atraumatic limps are commonly developed due to benign conditions such as toxic synovitis, in some cases the limp can occur as a manifestation of a serious or life-threatening condition [3].

Generally, a child develops a mature gait pattern by three years of age, and it consists of stance and swing phases [4]. Deviation from normal gait can be classified as antalgic and non-antalgic gaits. An antalgic gait results from pain in an affected limb, in which the stance phase becomes shorter. In contrast, several types of limping are associated with non-antalgic gait and most of them do not need urgent evaluation and treatment [5, 6]. The cause of limping usually can be determined via history, physical examination and necessitous laboratory and imaging investigations [4, 7]. Viral infections are a well-recognized cause of acute arthralgia and arthritis with a large number of causative agents. A careful consideration of epidemiological, clinical and serological properties are required for making the correct diagnosis. Multiple studies have suggested some viral infections, including Parvovirus B19, Hepatitis B and C, Human Immunodeficiency Virus (HIV), have a tendency to cause arthritis [8–10], but there are not any reports of the novel Coronavirus rheumatogenicity.

Here, we report two cases of gait disturbances following SARS-CoV-2 infection. Both cases were managed and treated successfully without any sequel.

## Case presentation

### Case-1

**Fig. 1:**
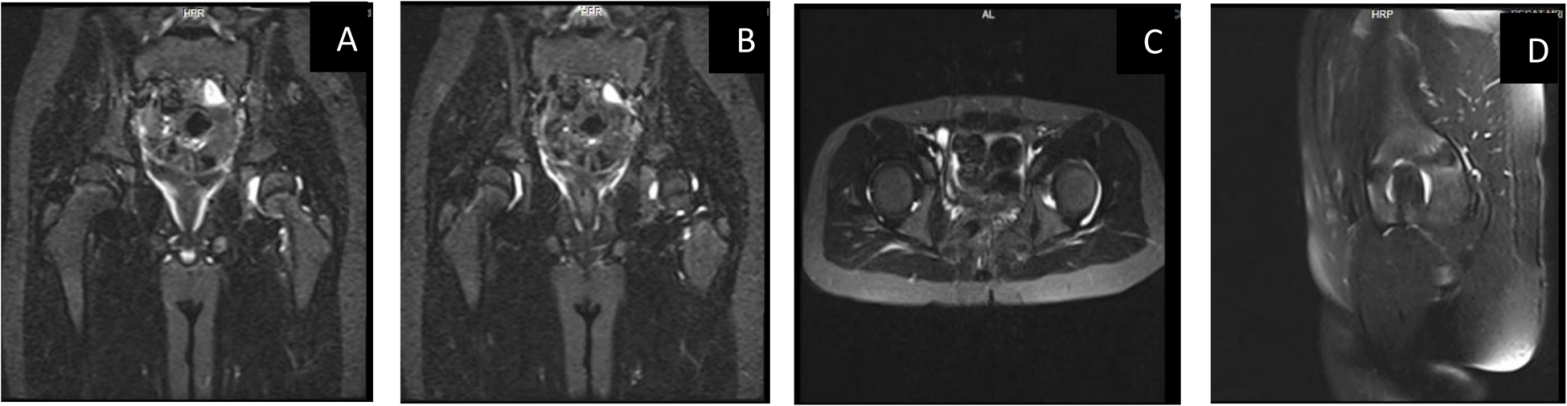
STIR sequencing MRI of both hip joints of the first patients. **a-b** Coronal view. **c** Axial view. **d** Sagittal view. Both hip joints effusion, especially in left side

An 8-year-old previously healthy boy was referred to the pediatric rheumatology center of Kerman University, in Southeast of Iran, for left side lower extremity gait disturbance and limping. This condition had appeared three weeks prior to his visit and he did not have any history of trauma or vigorous activity. However, he had experienced mild respiratory tract infection including low-grade fever and cough approximately one week before refusing to walk. During this time, he had visited an orthopedist. However, his condition was not diagnosed in that visit. Finally, he was referred to our clinic for more evaluations. At the time of his first visit, the patient did not have a fever. We performed a complete and systematic examination. He had an antalgic gait at his left side due to difficulty in weight bearing. His physical examination revealed pain and limitation in flexion, internal and external rotational movements of left hip with a positive FABER Test. There was not any evidence of other contributing factors and red flags, including organomegaly, lymphadenopathy, skin lesions, and neurological deficits. His laboratory evaluations revealed a White Blood Cell (WBC) count of 9$\times$10^9^ /L with an Absolute Neutrophil Count (ANC) of 3.2 $\times$10^9^ /L and an Absolute Lymphocyte Count (ALC) of $4.64\times$10^9^ /L, a platelet count of 312,000/µL, and a hemoglobin of 15.5 g/dl. The Erythrocyte Sediment Rate (ESR) and C-Reactive Protein (CRP) were 3mm/h and 3.5 mg/L, respectively (Table 1). Considering the preceding symptoms of fever and cough, a serological investigation for both IgM and IgG of SARS-CoV-2 infection was performed. The tests indicated a high IgM level of 3.4 (with a 1.1 cut off) in the first test, and a high IgG level of 3.8 (with a 1.1 cut off) in the second test, which was performed one week later. Detection of SARS-CoV-2 antibodies was performed using SARS-CoV-2 immunoglobulin M (IgM) ELISA kits (Pishtaz Teb, Iran, http://pishtazteb.com) and SARS-CoV-2 IgG ELISA kits (Pishtaz Teb, Iran http://pishtazteb.com) according to the manufacturer’s protocol (Specificity: 97.30 %; Sensitivity: 79.40 %) [11]. The nasopharyngeal swab for coronavirus Polymerase Chain Reaction (PCR) assay was not tested due to latency evaluation from preceding symptoms and patient’s parents refusal. There was not any evidence of marked joint space widening, reactive bone and fatty changes and other pathologic findings in his hip X-Ray. In addition, his chest radiography was normal. He underwent hip joints ultrasound study with a result of 7mm effusion in left side. Finally, Magnetic Resonance Imaging (MRI) showed a moderate joint effusion at his left hip articulation and a minimal joint effusion at his right hip joint (Fig. 1). Treatment was administered by skin traction and Naproxen 250 mg twice a day. The patient consequently recovered approximately one week later without any issues with either hip movement restriction or gait pattern and mobility.

**Table 1:** The Laboratory finding of both patients at the time of limp. The acute phase reactant of both patient were in normal range after 1 week

Laboratory Test	Case-1	Case-2
WBC(5-14.5)10*3/µL	9.3	9.7
RBC(3.9–5.3)10*6/ µL	6.16	4.11
ANC	3255	5600
ALC	5700	2650
Hb (11.5–15.5 g/dl)	15.9	11.9
MCV(75–87 fL)	75.6	82
Platelet(172–450)10*3/ µL	310	511
ESR(0–15 mm/h)	7.0	39.0
CRP(0–10 mg/l)	13.0	12.0
PBS for blast	Negative	Negative
BUN	10	7
Cr (0.5-1 mg/dl)	0.5	0.6
AST (5–60 IU/ml)	24	23
ALT (6–50 IU/ml)	17	12
ALP (180–1200 IU/ml	558	339
Ferritin(11–92 ng/ml)	N.A	49.30
LDH (< 746 U/L)	385	409
Uric Acid (3-6.4 mg/dl)	2.1	N.A
Wright Agglutination Test	Negative	Negative
2ME	Negative	Negative
Coombs Wright	Negative	Negative
ASO-Titer (up to 200 IU/ml)	62	31
ANA (IU/ml)	Negative (0.2)	Negative
RF (< 16 IU/ml)	27	Negative
Coronavirus IgM (< 1.1)* Indirect ELISA	3.8	1.2
Coronavirus IgG (< 1.1)** Indirect ELISA	3.4	0.35
Coronavirus PCR	N.A	Positive

* & **: Detection of SARS-CoV-2 antibodies was performed using SARS-CoV-2 immunoglobulin M (IgM) ELISA kits (Pishtaz Teb, Iran, http://pishtazteb.com) and SARS-CoV-2 IgG ELISA kits (Pishtaz Teb, Iran http://pishtazteb.com) according to the manufacturer’s protocol. Pishtaz Teb Zaman diagnostic Kits (Specificity: 97.30 %; Sensitivity: 79.40 %)

### Case-2

**Fig. 2:**
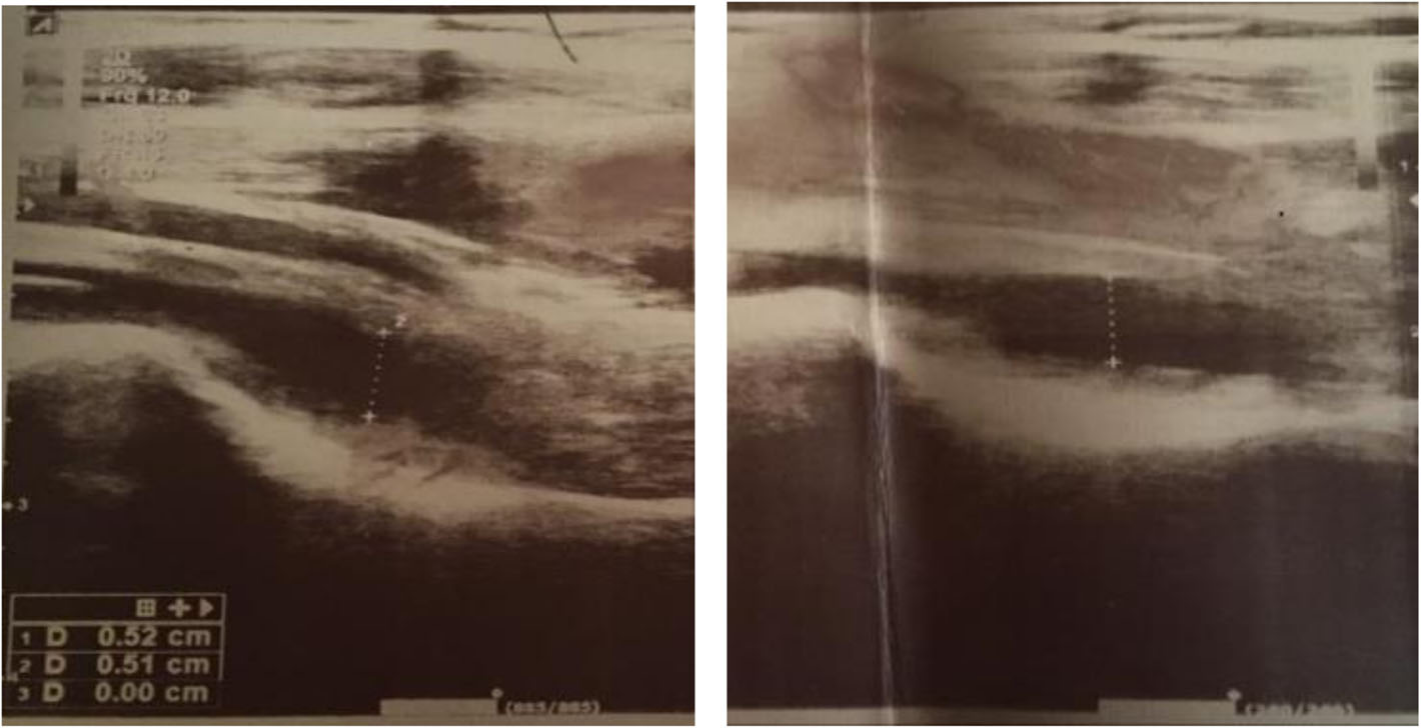
Ultrasound of both hip joints in the second patient. Bilateral mild hip joints effusion, especially in anterior synovial space of left side

A 6-year-old girl, who had experienced a high-grade fever for two days approximately one week prior to her visit, was referred to our outpatient Clinic due to her left side lower extremity limp. She complained of polyartheralgia in several joints including both knees and wrists with a predominant left lower limb pain in groin region and knee. She was born from a consanguineous marriage from a repeat cesarean section with a birth weight of 2.7 Kg. She had a history of right side hydronephrosis, which had gradually improved. The patient also had a history of limping three years ago, which had led to her hospitalization and aspiration of the right hip joint fluid. She had been unsuccessfully treated for septic arthritis by an orthopedic surgeon. Her mother is a nurse, who had been working in a secondary hospital facility for COVID-19 patients. At the time visit, the patient was thoroughly examined; she did not have fever and her vital signs were within the normal range. She could not bear weight on her lower left limb and had an antalgic gait. During examination, she showed no evidence of muscle involvement, but she had some limitations in the range of movements of her left hip joint and scant tenderness in both wrists. Despite the hip joint involvement, she did not have any compensatory gait pattern, any other limitations such as knee flexion, or any remarkable foot and ankle positioning. Her laboratory evaluation were as followings: WBC: 9.68$\times$10^9^ /L (Neutrophils = 5.69 $\times$10^9^ /L & Lymphocyte = $2.65\times$10 ^9^ /L), platelet count: 511,000/µL, Hemoglobin: 11.9 g/dl, ESR = 39mm/h and CRP = 12 mg/L. She also tested positive for Coronavirus using PCR analysis. Serological investigation for both IgM and IgG of SARS-CoV-2 infection revealed a mild increase in IgM level to 1.2 (with a 1.1 cut off) without increasing the IgG level [11] (Table 1). Further hip X-Ray was normal. The hip ultrasound showed evidence of mild joint effusion in both hip joints especially in the anterior part of her left side (Fig. 2). This patient was also diagnosed with post coronavirus reactive arthritis, and successfully treated with Ibuprofen 40 mg/kg/day administered in three doses during the day. She completely recovered and her laboratory and ultrasound findings became normal after 4 days. Her follow up at least for 45 days later did not show any subsequent signs or sequels.

## Discussion and conclusions

Preliminary reports of SARS-CoV-2 infection suggest less frequent and severe presentation of the illness in children [12]. As COVID-19 continues to spread, there is growing evidence that children may be vulnerable to another rare or even a serious complication of the disease. In late April 2020, European countries released several warnings after emergence of severe infections caused by SARS-CoV-2. These were then followed by reports of this infection from New York and other parts of the United States [13]. The features of this multisystem inflammatory syndrome (MIS-C) which has a wide range of demonstrations, some of which are known such as Kawasaki Disease (KD), Toxic Shock Syndrome (TSS), Kawasaki Shock Syndrome, and secondary hemophagocytic lymphohistiocytosis/ macrophage-activated syndrome, are astonishing. However, they seem to be due to a late immune effect [12].

Reports of viral associated arthritis in this pandemic are lacking in pediatric patients. The diagnosis of this condition can be difficult. Serological tests integrate both clinical and epidemiological data. Accurate data regarding the incidence and prevalence of viral induced arthritis are insufficient. Studies have suggested a viral etiology in about 1 % of cases of acute arthritis. Parvovirus B-19, Hepatitis-B and C, alpha viruses and HIV are among the most important causes of virally induced arthritis [14].

The pathogenesis of post viral arthritis is complex. First, they often do not meet the classical definition criteria of reactive arthritis. Second, during some viral infections (e.g. rubella, varicella zoster, herpes simplex virus, cytomegal virus, etc.) the virus can be isolated in synovial fluid. Third, in some viral infections (e.g. hepatitis-B, adenovirus type-7), antigen-antibody immune complexes were isolated from synovial fluid, highlighting a their possible role in the pathogenesis of virally induced arthritis [14–17]. Generally, post infectious arthritis affects the lower limb joints especially ankles and knees [18, 19]. However, we should note that in approximately 50 % of cases the etiologic agent cannot be isolated, and in 25 % of children with post infectious arthritis the infection is asymptomatic [15].

Our patients neither had history of falling, other traumatic injuries nor showed evidence of serious and life-threatening conditions such as septic arthritis, osteomyelitis, malignancies, and other possibilities. Both had first shown mild symptoms of upper respiratory infection including fever and cough approximately seven days prior to limping. The first case had a prolonged duration of limping for three weeks, and the second case showed polyartheralgia in addition to limping. The first patient, had significantly higher than normal levels of IgM and IgG against SARS-CoV-2 at the time of visit and after two week. The patient’s parents did not consent to performing a nasopharyngeal PCR test. However, we believe that it would have been of little value because of the delay in the patient’s visit. The patient was diagnosed with reactive arthritis and responded well to the NSAID treatment. After two months, he was completely well and had no remaining problems. The second patient, tested positive for both serological and molecular tests. While she had a positive PCR test after her mild fever and cough one week prior to her visit, she had only a marginally higher than normal level of IgM a normal level of IgG at the time of her limp, illustrating the possibility of a more direct impact of SARS-CoV-2 on the joints. These two patients demostrate the coronavirus arthrogenicity, which has been reported numerously for other viral infections, but has not yet been reported for SARS-CoV-2 infection in children. Nowadays, we see more cases of coronavirus related arthralgia, requiring more attention of clinicians especially pediatricians to these aspects of COVID-19. Further studies are required for characterization of the coronavirus arthrogenicity and rheumatogenicity. In addition, analysis of intra-articular specimen will help characterize these impacts of SARS-CoV-2.

## Data Availability

All data generated or analyzed during this study are included in this published article [and its supplementary information files].

## Abbreviations

NSAID: Non-Steroidal Anti-Inflammatory Drugs
HIV: Human Immunodeficiency Virus
WBC: White blood cell
ANC: Absolute Neutrophil count
ALC: Absolute Lymphocyte count
RBC: Red Blood Cells
MRI: Magnetic resonance imaging
PCR: Polymerase Chain Reaction
